# Music and emotion in Alzheimer’s disease

**DOI:** 10.1186/s13195-019-0523-y

**Published:** 2019-08-07

**Authors:** Eva M. Arroyo-Anlló, Stéphanie Dauphin, M. Noelle Fargeau, Pierre Ingrand, Roger Gil

**Affiliations:** 1 Department of Psychobiology, Neuroscience Institute of Castilla-León, University of Salamanca, Av. de la Merced s/n 37007, Salamanca, Spain; 2 Department of Neurology Faculty of Medicine, University Hospital, CHU La Milétrie, 2, Rue de la Milétrie, 86021 Poitiers, France; 3 Department of Biostatistics Faculty of Medicine, University of Poitiers, 2, Rue de la Milétrie, 86021 Poitiers, France; 4 Emeriti Professor of Neurology, University Hospital, Poitiers, France

**Keywords:** Emotion, Music, Dementia, Memory, Prosody

## Abstract

**Background:**

Alzheimer’s disease may compromise several musical competences, though no clear data is available in the scientific literature. Furthermore, music is capable of communicating basic emotions, but little is known about the emotional aspect of music in patients with Alzheimer’s disease. We present a systematic investigation of music processing in relation to extra-musical skills, in particular emotional skills in patients with Alzheimer’s disease.

**Methods:**

We tested 30 patients with mild or moderate Alzheimer’s disease and 30 control subjects. We essentially evaluated (a) musical competences, using the extra-linguistic test, Solfeggio test and the recognition test of musical emotions—elaborated by our research team—and the Seashore test, and (b) emotional capacities using emotional memory and emotional prosody tests—made by our research group.

**Results:**

We significantly observed lower total results of every test assessing cognitive, emotional and music competences in Alzheimer’s disease patients than those in control subjects, but the score of musical emotion recognition test did not reach to a significant difference between the subjects groups.

**Conclusions:**

Our findings found a global impairment of music competences in Alzheimer patients with cognitive and emotional troubles. Nevertheless, the performances in the recognition test of musical emotions showed a trend towards a performance difference. We can suggest that Alzheimer’s disease currently presents an aphaso-agnoso-apractic-amusia syndrome.

## Introduction

The capacity to perceive, experience and produce music is a fundamentally human characteristic, present universally across different cultures [1]. Music needs a complex information processing, requiring the analysis of a musical stimulus target against the acoustic background, representation of the musical source (instrumental or vocal timbre) and tracking of pitch (melody) and temporal (rhythm, metre) information, as well as music memory (for review, see [2]).

Alzheimer’s disease (AD) is a degenerative neuropathology characterized by a progressive decline in cognitive and behavioural functions, due to a progressive posterior atrophy, in particular of medial temporal lobes. AD may compromise several musical competences, though no clear data are available in the scientific literature. Several studies have found impaired tone recognition [3], pitch perception and tonal working memory [4–9], timbre [10] or rhythm [11] in AD patients, but other studies have observed relatively preserved ability to detect basic acoustic changes in music as pitch [10, 12, 13], timbre or rhythm [8, 14].

Concerning musical memory in AD, most studies have observed relatively spared with familiar music [13, 15–19], depending more on semantic musical memory. In contrast, heterogeneous results have been found using unfamiliar music [8, 19–25], exploring more episodic musical memory.

Furthermore, music is capable of communicating basic emotions [26], which are recognized effortlessly in adults and regardless of musical training [27]. Despite much recent interest in music and emotion, little is known about emotional aspect of the music in patients with AD. In contrast, recognition for facial, visual or vocal expressions of emotion has been more studied in AD [27–29]. In general, most studies on emotion in AD have observed that emotion is not completely abolished, because patients are able to remember better the words with emotional valence than neutral ones or to remember better autobiographical events with greater emotional charge [30].

Concerning musical emotions, studies with AD patients have found that they can perceive and recognize emotions conveyed by music as well as recognize the melodies and titles of familiar songs [4, 12, 13, 31, 32]. Familiar music has an enormous power to evoke personal emotions and memories [32]. Several neuroimaging studies in dementia groups have found that impaired recognition of musical emotions appears to be related to the degree of atrophy specifically in the anterior temporal lobe, which is more common in semantic dementia than in AD [12, 13, 33, 34].

In order to let us understand the relations between musical and emotional competences in AD, we present a systematic investigation of music processing in relation to extra-musical skill, in particular emotional skills in patients with Alzheimer’s disease.

## Material and methods

### Subjects

We studied 60 subjects, including 30 patients diagnosed with Alzheimer’s disease and 30 normal healthy controls. All participants were native French speakers and interested in music, but not music professionals. They were right handed and had no hearing problems.

Thirty patients with a clinical diagnosis of probable Alzheimer’s disease were recruited at the Department of Neurology and Neuropsychology, University Hospital, CHU La Milétrie at Poitiers, France. They lived at home with a family caregiver. All AD patients had a history of progressive decline in intellectual function without focal motor or sensory features. To exclude other possible causes of dementia, appropriate laboratory tests were performed and these gave normal results. No findings incompatible with a diagnosis of AD were found in the electroencephalogram, electrocardiogram or chest X-ray in any of the patients. Brain CT scan revealed mild cortical and central atrophy, but no other pathology. All Alzheimer patients met the diagnostic criteria of the “NINCDS - ADRDA Work Group” and of the DSM-5. All had a score of less than 5 on the Hachinski Ischemic Scale. According to the Clinical Dementia Rating System (CDR) [35] and Mini-Mental State Examination (MMS), 13 patients had mild AD and 17 patients had moderate AD. All AD subjects were taking anti-dementia medication during the study, in particular anti-acetyl cholinesterase treatments. Only the AD patients whose verbal comprehension (assessed by the MMS three-stage command) were equal to or above 2 were included in the study.

Controls primarily comprised of volunteers from French community or were members or visitors at the University Hospital in Poitiers (France) whose results in a neurological examination were normal, had a Clinical Dementia Rating Scale (CDR) equal to 0 and scored 28/30 or higher on the Mini-Mental Status Examination (MMS).

### Material

Neuropsychological assessment consisted of testing (a) cognitive and mood states, as well as emotional capacities, and (b) musical competences.

A. Cognitive and emotional assessments

On the one hand, we used the Mini-Mental State Examination (MMS) to assess cognitive aspects and The Hospital Anxiety and Depression Scale (HAD) of Zigmond y Snaith [36] for anxiety-depression evaluation.

On the other hand, we assessed the emotional capacities using two tests elaborated by our research team: (a) emotional memory test and (b) test of emotional prosody. Concerning the first test evaluating emotional memory, it consisted of the learning of two lists of words, one of sixteen words with negative emotional connotation (list A: e.g. corpse, disease...) which are matched in length and frequency with another list of sixteen neutral emotional words (list B: e.g. place, shape...). The goal is to compare the learning of an emotionally negative word list to learning emotionally neutral word list, in order to understand the impact of the emotion aspect on episodic learning. We ask them to learn each list of words over five successive trials (assessing the encoding ability and learning) and a long delay free recall is made 20 min later. The emotional memory score reflects the impact of the words emotion on learning and it was the words’ total of five trials in list B minus the total of the five trials from list A.

Concerning the test of emotional prosody, it is composed of three subtests, evaluating the spontaneously intonation expression, repetition of an intonation and recognition of emotional prosody, using the recorded sentences. Each sentence was recorded technically with Apple’s Logic Pro 8 and was presented via over-ear ATH-M30 professional headphones from Audio-Technica (http://www.audio-technica.com) in the same order for all participants. In the absence of an objective measure of auditory acuity, participants were asked during a pre-evaluation example to adjust the volume to a comfortable and clearly audible level. In stimulus presentation, the minimum selected headphone volume proved to be between 65 and 70 decibels (dB) and the maximum between 80 and 85 dB. The first subtest consisted of spontaneously producing a sentence with a proper emotion in function of the context. We used four kinds of emotions: joy, sadness, anger and neutral (e.g. The attitude of an employee is unacceptable, the boss tells him [context]: “I’m fed up and more than fed up, next time it’s the door”). The second subtest consisted on intonation repetition of a sentence, using three semantically neutral sentences presented with four different intonations—joy, sadness, anger and neutral. The third subtest consisted of the identification of the sentence emotion (joy, sadness, anger and neutral). We used 12 sentences, three for each emotion type and each sentence could be heard twice, but we considered the first answer done by subject to score. All responses were recorded to assess later for independent examiners. The criteria for the rating of the first two subtests was carefully determined by two of the examiners as perfect (two points), partly perfect (one point) and incorrect (no points). The maximum score of each sentence was one point across the test and the maximum scores of each subtest were 12 points, respectively.

B. Musical competences’ assessment

We evaluated the musical competences using four tests: extra-linguistic test, Solfeggio test, recognition test of musical emotions and Seashore test [37]. The first three tests were elaborated by our research team, taking account of the western tonal system. The selected music tracks are listened to for about a minute and the response time is 1 min maximum.

Concerning the extra-linguistic test, it assessed three aspects: (a) recognition of six rhythmic structures, (b) eight musical items of familiar and unfamiliar melodic memory and (c) recognition of five musical timbres. Firstly, participants had to identify the musical style that they have recognized in a total of six extracts listened to—one extract per musical style (Tango/Waltz/Paso doble/Slow/Walk/Rock). Secondly, subjects had to identify and repeat the total of five very traditional French songs (e.g. “Mon beau sapin”/“Les rois mages”…). They had to say the title of the familiar melody they think they had recognized, because they previously sang or hummed this in the air. After that, they must identify one melody which is not familiar, which has been previously listened to, among two other melodies. And thirdly, all subjects must recognize a total of five different musical timbres and name the musical instrument they recognized in the musical excerpt broadcast (Piano/Guitar/Organ/Hunting Horn/Violin). The maximum total score of the test was 19 points, a point for each correct musical item.

Concerning the Solfeggio test, it was composed of two subtests: the recognition of twenty notes and the writing of fifteen notes. The maximum score of each correct note was one point and the total score was 35 points.

In addition, we also performed the Seashore test (for instructions and items, see [37]), in order to also evaluate the musical competences. This test contains six measurements for which we take only the first ten items of each subtest as follows: (a) sense of pitch interval (10 pairs of tones differing in frequency from 17 to 2 Hz; the subject was asked whether the second tone is higher or lower than the first); (b) sense of loudness (10 pairs of tones differing in intensity from 4.0 to 0.5 dB; the subject was asked whether the second tone was stronger or weaker than the first); (c) sense of rhythm (10 pairs of rhythmic patterns; the subject was asked whether they are the same or different); (d) sense of time (10 pairs of tones differing in duration by 0.3 to 0.5 s; the subject was asked whether the second was longer or shorter than the first); (e) sense of timbre (10 pairs of tones, each of which was made up of the fundamental first five harmonic intervals with the intensities of the third and fourth harmonies being varied; the subject was asked whether they were the same or different; and (f) tonal memory (10 pairs of tone sequences with one tone different in the second sequence as compared to the first; the subject was asked which tone is different). The score was one point per exact answer, and the maximum total score was 60 points.

Regarding the musical emotion recognition test, we previously proposed to recognize the emotion evoked by 12 extracts of musical pieces (4 pieces per emotion—joy, fear or sadness) to 84 young people, in order to choose the extracts, whose emotion was clearly recognized by more than 90% of them. For that, we used the following opened question, in order to allow them to freely communicate the feelings evoked by each musical piece: “How do you feel by listening to these music excerpts?”. We noted for each musical extract, the words that inspired them and when the emotion was clearly recognized, the notation was joy, fear or sadness. Thus, we could only select 6 musical extracts according to three primary emotions—2 extracts per emotion: (a) joy (“The Four Seasons—Spring” by Vivaldi and “Folies Bergère” from Paul Lincke, which have been recognized 100% as joy emotion by the young group), (b) sadness (“The Funeral March” of Chopin and “The Dispute” of Yann Tiersen: 90% and 97%, respectively) and (c) fear (“The theme of Psychosis” by Bernard Herrmann and “The theme of Teeth of the Sea” by John Williams: 97%). After that, we used those six musical extracts in the musical emotion recognition test. All participant groups had the following instruction for each musical extract: “Choose—on the sheet—the emotion in which it makes him think of joy-sadness-fear”. The maximum score was 6 points (one point per correct answer).

### Procedure

The assessment was carried out along a one-week period in 2 sessions, each one in two different days. The first session was for the evaluation of cognition, mood and emotional capacities, and the second one was exclusively for assessing the musical competences. All participants were tested individually in two sessions with the same order of testing. Each session took between 45 and 60 min to complete.

### Statistical analyses

Data was analysed using the Statistical Package SAS—software version 9.2. In this study, an *α* level of 0.05 was selected for statistical significance. To find significant differences in gender and laterality distributions among the groups, we used the non-parametric test of Fisher, and in age, mood state, educational and musical level, we used the non-parametric test of Kruskal-Wallis.

To compare the mean scores of musical and emotional capacities between participant groups, the Wilcoxon or Kruskal-Wallis test was used. We also used Spearman’s correlation analysis to analyse the relationships between the music competences and cognitive-emotional aspects.

## Results

### Demographic characteristics

Sixty subjects participated in the study: two experimental groups, one of 13 patients with mild AD (MiAD) and another with 17 patients had moderate AD (MoAD), and a control group of 30 healthy subjects.

Concerning the experimental AD groups (19 women and 11 men), the mean duration of the disease was 3.2 years for MiAD and 4.9 years for MoAD. The MiAD group (7 women and 6 men) had a mean age (± SD) of 74.8 years old (± 3.58), a MMS score (± SD) of 23.8 (± 2.91) and a mean educational level (± SD) of 4.12 (± 0.39) using the classification of Barbizet and Duizabo [38]: illiterate (educational level (EL) 1), able to read, to write and to count (EL 2), 6 years of education (EL 3), 9 years of education (EL 4), 11 or 12 years of education (EL 5), 13 years of education (EL 6) and more than 13 years of education (EL 7). The MiAD patients had a mean musical level (± SD) of 1.30 (± 0.15) using the classification of Sol [39]: no musical learning or playing instrument (musical level (ML)1), music theory learning or playing instrument for 1 year (ML2), regular music theory learning or playing instrument for 2 to 5 years (ML3), regular music theory learning or playing instrument for 6 to 10 years (ML4), high music studies and/or performed in concert (ML5), currently performing live in concerts (ML6). The MoAD group (12 women and 5 men) had a mean age (± SD) of 76.1 years old (± 2.93), a MMS score (± SD) of 14.9 (± 2.20), a mean educational level (± SD) of 3.97 (± 0.61) using the classification of Barbizet and Duizabo [38] and a mean musical level (± SD) of 1.51 (± 0.11) using the classification of Sol [40].

Concerning the control group (18 women and 12 men), the mean age (± SD) was of 75.9 years old (± 1.30). It had a MMS score (± SD) of 28.6 (± 1.22), a mean educational level (± SD) of 3.99 (± 0.24) using the classification of Barbizet and Duizabo [38] and a mean musical level (± SD) of 1.21 (± 0.30) using the classification of Sol [39].

There were no significant differences in gender (*p* = 0.56), laterality (*p* = 1), age (*p* = 0.14) and educational (*p* = 0.40) and musical (*p* = 0.78) level distributions among the groups. The clinical and demographic data of the participating groups are shown in Table 1.

**Table 1:** Means (M) and standard deviations (SD) of several demographic and clinical characteristics of the AD and control groups

	MiAD group	MoAD group	Control group	
	M (SD)	M (SD)	M (SD)	*p* value^a^
Sample size (*N*)	13	17	30	
Gender (M:F)	6:7	5:12	12:18	> .05
Age (years)	74.80 (3.58)	76.11 (2.93)	75.92 (1.30)	> .05
Educational level	4.12 (0.39)	3.97 (0.61)	3.99 (0.24)	> .05
Musical level	1.30 (0.15)	1.51 (0.11)	1.21 (0.30)	> .05
Mini-Mental State	23.80 (2.91)	14.90 (2.20)	28.60 (1.22)	< .05*
Hospital Anxiety and Depression Scale	Anxiety: 5.2 (2.9) Depression: 3.5 (2.4)	Anxiety: 5.2 (4.2) Depression: 3.2 (1.8)	Anxiety: 6.6 (2.8) Depression: 3.4 (1.8)	> .05

^a^*p* values referred to control and experimental groups

*Significant difference (*p* < .05)

### Cognitive and emotional aspects

Concerning cognitive state (Table 1), we found a significant difference between three participant groups for MMS score (*p* = 0.0018), as well as between the MiAD and MoAD groups (*p* = 0.007). In regard to mood state assessed by the HAD test, the MiAD group had an anxiety subtest score (± SD) of 5.2 (± 2.9) and a depression subtest score of 3.5 (± 2.4) and the MoAD group had an anxiety subtest score (± SD) of 5.2 (± 4.2) and a depression subtest score of 3.2 (± 1.8), and so, the control group had an anxiety subtest score (± SD) of 6.6 (± 2.8) and a depression subtest score of 3.4 (± 1.8). All groups did not differ significantly between them (anxiety subtest: *p* = 0.18; depression subtest: *p* = 0.62).

Regarding the emotional capacities, the analysis of the results shows a significant difference between the three groups for the emotional memory test (*p* < 0.0001; Table 2), both for the list of emotionally charged words (list A) and for the list of emotionally neutral words (list B). All AD patients had lower scores for every word list learning and long delay free recall than the control group. We also observed a significant difference of emotional memory score between all participant groups (*p* = 0.0086), as well as between the control and MiAD groups (*p* = 0.012), but not between AD groups (*p* = 0.068) for the emotional memory (Table 2). Wilcoxon analysis showed that learning and delayed recall performances were worse between the control group and MiAD group (*p* ≤ 0.0002). However, MiAD had better learning than MoAD, but the long delay free recall performances are similar between both groups (*p* > 0.05; Table 2).

**Table 2:** Means (M) and standard deviations (SD) of both emotional capacity tests (emotional memory and emotional prosody tests) in the AD and control groups

	MiAD group	MoAD group	Control group	
	M (SD)	M (SD)	M (SD)	*p* value^a^
Emotional memory test				
Total learning of emotionally charged word list (list A)	26.3 (5.5)	17.7 (6.5)	47.4 (13.2)	< 0.0001*
Total learning of emotionally neutral word list (list B)	21.1 (5.1)	14.5 (5.1)	38.4 (14.1)	< 0.0001*
Long delay free recall of list A	2.2 (2.2)	0.8 (0.9)	10.0 (3.6)	< 0.0001*
Long delay free recall of list B	0.8 (1.6)	0.2 (0.8)	7.3 (4.0)	< 0.0001*
Emotional memory score	5.2 (5.7)	3.2 (4.5)	9.0 (7.9)	0.0086*
Emotional prosody test				
Spontaneously intonation expression	9.6 (2.2)	8.5 (2.2)	11.1 (1.1)	0.0005*
Intonation repetition	29.4 (5.9)	14.5 (5.1)	33.1 (3.8)	0.0036*
Recognition of sentence emotion	8.6 (2.4)	7.8 (2.1)	9.8 (2.6)	0.0185*

^a^*p* values (Kruskal-Wallis test) referred to control and experimental groups

*Significant difference (*p* < .05)

There was a significant difference for the emotional prosody test between the three participant groups (Table 2). The performances of every subtest of this emotional prosody test were statistically lower in AD groups than those of the control group (spontaneous intonation expression: *p* = 0.0005; intonation repetition: *p* = 0.0036 and recognition of sentence emotion: *p* = 0.0185). However, the Wilcoxon test showed only significant difference for intonation spontaneous expression between the MiAD and control groups (*p* = 0.0230).

### Musical competences

**Fig. 1:**
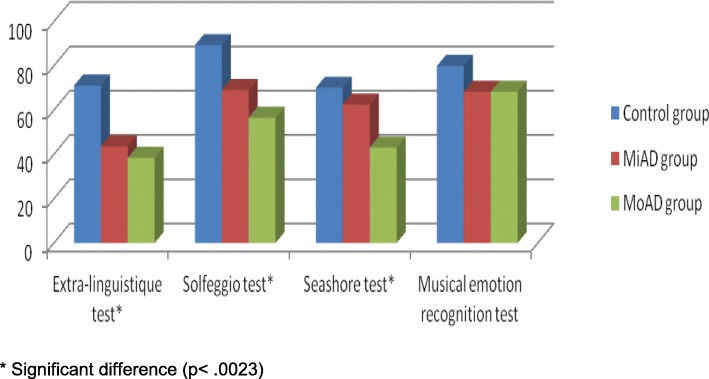
Percentages of good answers in musical competences’ tests for the AD and control groups. *Significant difference (*p* < .0023)

In general, we significantly found lower total scores of every test assessing music competences in AD patients than those in control subjects, except for the score of musical emotion recognition test which did not reach to a significant difference between the subject groups (Fig. 1 and Table 3).

**Table 3:** Means (M), percentages and standard deviations (SD) of the extra-linguistic, Solfeggio and Seashore tests as well as the musical emotion recognition test in the AD and control groups

	MiAD group	MoAD group	Control group	
	M ± SD (% ± %SD)	M ± SD (% ± %SD)	M ± SD (% ± %SD)	*p* value^a^
Extra-linguistic test				
Recognition of rhythmic structures	1.9 ± 1.4 (31.7 ± 23.3)	1.6 ± 1.2 (26.7 ± 20)	3.9 ± 1.9 (65 ± 31.7)	0.0013*
Familiar melody memory	2.4 ± 1.2 (48 ± 24)	2.6 ± 1.1 (52 ± 22)	3.4 ± 1.0 (68 ± 20)	0.0126*
No familiar melody recognition	1.5 ± 0.8 (50 ± 26.7)	1.1 ± 1.0 (36.7 ± 33.3)	2.4 ± 0.7 (80 ± 23.3)	0.0002*
Recognition of musical timbres	2.4 ± 1.4 (48 ± 28)	2.0 ± 1.4 (40 ± 28)	3.7 ± 1.5 (74 ± 30)	0.0025*
Total score	8.3 ± 3.7 (43.6 ± 19.5)	7.3 ± 3.0 (38.4 ± 15.8)	13.5 ± 4.3 (71.1 ± 22.6)	0.0002*
Solfeggio test				
Notes recognition	15.3 ± 6.9 (72.8 ± 32.9)	13.9 ± 7.4 (66.2 ± 35.2)	18.7 ± 4.3 (89 ± 6.2)	0.1370 ns
Notes writing	8.8 ± 4.8 (62.9 ± 34.3)	5.9 ± 5.0 (42.1 ± 35.7)	12.6 ± 1.7 (90 ± 12.1)	0.0001*
Total score	24.2 ± 10.7 (69.1 ± 30.6)	19.8 ± 11.7 (56.6 ± 33.4)	31.3 ± 5.3 (89.4 ± 15.1)	0.0022*
Seashore test				
Pitch	5.5 ± 2.4 (55 ± 24)	4.4 ± 2.2 (44 ± 22)	6.5 ± 2.4 (65 ± 24)	0.0610 ns
Loudness	6.2 ± 2.5 (62 ± 25)	4.4 ± 2.9 (44 ± 29)	7.5 ± 2 (75 ± 20)	0.0027*
Rhythm	7.3 ± 2.7 (73 ± 27)	5.5 ± 2.4 (55 ± 24)	7.9 ± 1.9 (79 ± 19)	0.0089*
Time	7.7 ± 1.7 (77 ± 17)	5.2 ± 2.7 (52 ± 27)	7.7 ± 2 (77 ± 20)	0.0043*
Timbre	6.8 ± 1.6 (68 ± 16)	4.6 ± 2.4 (46 ± 24)	6.6 ± 2 (66 ± 20)	0.0090*
Tonal memory	3.9 ± 2.3 (39 ± 23)	2.1 ± 1.9 (21 ± 19)	6 ± 3 .2 (60 ± 32)	0.0008*
Total score	37.5 ± 6.4 (62.5 ± 10.7)	25.9 ± 10.2 (43.1 ± 17)	42.1 ± 9.3 (70.1 ± 15.5)	< 0.0001*
Musical emotion recognition test	4.1 ± 1.3 (68.3 ± 21.6)	4.1 ± 0.8 (68.3 ± 13.3)	4.8 ± 1.4 (80 ± 23.3)	0.0634 ns

^a^*p* values (Kruskal-Wallis test) referred to control and experimental groups

*Significant difference (*p* < .05)

*ns* no significant difference

There were significant differences in total scores of extra-linguistic and Solfeggio tests between the 3 participant groups. The extra-linguistic performances of the MiAD and MoAD groups showed more impairment to that of the control group (MiAD and MoAD: mean ± standard deviations: 8.3 ± 3.7 and 7.3 ± 3.0, respectively vs. 13.5 ± 4.3; *p* = 0.0002), as well as in the Solfeggio test (MiAD and MoAD: mean ± standard deviations: 24.2 ± 10.7 and 19.8 ± 11.7, respectively vs. 31.3 ± 5.3; *p* = 0.0022). In Table 3, it may be seen that significant differences were found in every aspect of the extra-linguistic test (rhythm recognition, melody memory and timbre recognition) between the three participant groups (*p* = 0.0013, *p* < 0.013 and *p* = 0.0025, respectively). However, we did not observe a significant difference in the note recognition subtest of the Solfeggio test between the subject groups (*p* = 0.137), but it was found in the notes written subtest (*p* = 0.0001; Table 3). Furthermore, the extra-linguistic test significantly distinguished MiAD from control participants (*p* = 0.0027), but not MiAD from MoAD patients (*p* = 0.3560). In contrast, the Solfeggio test did not significantly differ between the MiAD and control subjects (*p* = 0.0552).

All AD patients also had worse performances in the Seashore test than the control group (MiAD and MoAD: mean ± standard deviations: 37.5 ± 6.4 and 25.9 ± 10.2, respectively vs. 42.1 ± 9.3; *p* < 0.0001) and in its subtests, except for that of pitch sense (*p* = 0.061); (Table 3). However, we did not find significant differences in the Seashore test between the MiAD and control groups (*p* > 0.07). But the Seashore test showed statistically significant differences between the MiAD and MoAD patients in most of the Seashore subtests, except for pitch sense again (rhythm: *p* = 0.0393; time: *p* = 0.0068; timbre: *p* = 0.0081; tonal memory: *p* = 0.0340), as well as in its total score (*p* = 0.0019). Furthermore, we found a significant correlation between the extra-linguistic and Seashore tests in all participant groups (*r* = 0.416; *p* = 0.022). But considering the three shared items in both music tests (rhythm, timbre and tonal memory), only the timbre item showed an important correlation (*r* = 0.444; *p* = 0.013). Besides, significant correlations were found between the Seashore and MMS tests (*r* = 0.595; *p* = 0.0005), sentence emotion recognition of the emotional prosody test (*r* = 0.421; *p* = 0.020) and the emotional memory test, but only for the emotional word list—list A (*r* > 0.505; *p* < 0.0045). Besides, the Solfeggio test had also a significant correlation to recognition of sentence emotion of the emotional prosody test (*r* = 0.524; *p* = 0.0029), as well as the list A learning (*r* = 0.399; *p* = 0.0287). We also found a significant correlation between the extra-linguistic score and intonation repetition of the emotional prosody test (*r* = 0.406; *p* = 0.025), as well as the list A learning (*r* = 0.448; *p* = 0.012).

**Fig. 2:**
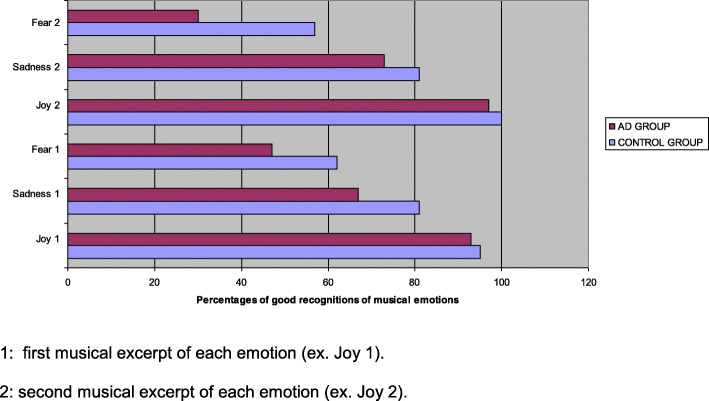
Percentages of good recognition of musical emotions in music excerpts produced by the AD and control groups. 1: First musical excerpt of each emotion (e.g. Joy 1). 2: Second musical excerpt of each emotion (e.g. Joy 2)

Concerning the musical emotion recognition test (Fig. 1), the total score of the whole AD group was lower than that of the control group, without reaching to significant difference (*p* = 0.0634). But the performances were similar between the MiAD vs. MoAD groups (mean ± standard deviations: 4.1 ± 1.3 vs. 4.1 ± 0.8, *p* = 0.9826) and MiAD vs. control group (mean ± standard deviations: 4.1 ± 1.3 vs. 4.8 ± 1.4, *p* = 0.1187). Considering each kind of primary emotions (joy, sadness and fear; Fig. 2), all AD patients recognized the joy emotion of musical excerpts as well as control participants (two joy musical excerpts: 93% and 97% vs. 95% and 100%) and something less the sad emotion of music (67% and 73% vs. 81% and 81%). However, the fear emotion of music was more difficult to recognize by AD patients than by the control group (47% and 30% vs. 62% and 57%).

## Discussion

The main goal of this study was to evaluate the music processing in relation particularly to emotional skill in AD, using a musical protocol including musical skills’ tests (extra-linguistic, Solfeggio and Seashore tests) and a musical emotion recognition test. Thus, we compared the music competences between two groups of patients with AD—MIAD and MoAD—and a control subject group, which they did not significantly differ in gender, laterality, age, educational and musical levels, as well as mood state, but AD patients showed the important cognitive and emotional deficits.

In general, the findings seem to suggest that global weakening of musical processing is a common disorder in AD. The results showed the lower performances of musical competences in AD groups than in the control group, assessing by the extra-linguistic, Solfeggio and Seashore tests.

The extra-linguistic performances were altered in MiAD patients, but it did not significantly decrease over the disease severity. The extra-linguistic aspects (recognition of rhythmic structures, melody memory and recognition of musical timbres) essentially put into play the capacities of working and long-term memory, reflecting the classical results about more important progressive deficit of short-term memory and learning than autobiographical memory [40]. We found the important verbal learning deficit and cognitive impairment in AD groups, though we did not include an auditory working memory test in our study which could have improved our results analysis. Several works have demonstrated that music memory is relatively preserved in patients with moderate to severe AD in spite of otherwise severe overall impairment [16, 17]. However, some reports have described impaired music memory in AD [20, 21, 23]. Besides, several studies have showed that recognition memory was better for the novel sung than spoken lyrics in AD patients [22, 24, 25]. Familiarity of music also is another aspect taken into account to explain the variability of music memory in AD patients [2, 18, 33, 41, 42]. In this sense, the recent study of Slaterry et al. [42] found out that in AD patients, unfamiliar melodies depended more on episodic memory, involving disease-associated activation group differences in precuneus and posterior cingulate cortex, and familiar melodies depended more on semantic memory, involving activation differences in right inferior frontal cortex. In addition, Baird and Samson [18] suggested that perhaps the implicit and procedural memories for musical stimuli remain preserved, but not musical episodic memory. Regarding neural basis, emotion and implicit memory share neural subcortical structures, such as the amygdales or basal ganglia, which are ontogenetically older than those of explicit memories. Furthermore, we found important and progressive deficits of the word lists learning in AD patients, more with the emotionally neutral word list than that with emotionally charged words. We also noted a positive emotional impact of words on learning (emotional memory score) in AD groups, despite the difficulties of memory. However, the emotional effect on learning was less important than that of the control group.

Concerning the Solfeggio test, we observed similar results for note recognition subtest between the participant groups, but not for the note writing subtest. Similar results were found by Sol [39]. This result could be explained in terms of the task difficulty degree. The cognitive competences of the note recognition subtest are similar to those of language recognition tasks, but not for note written subtest. In this last subtest, patients rewrote the name of the note in words on the music sheet instead of drawing it or they randomly placed notes on the music sheet. Nevertheless, we must also consider the praxis, spatial organization or understanding troubles in AD patients, explaining those results, but we did not find a relationship between MMS and Solfeggio scores.

However, we only found a significant correlation between MMS and Seashore scores, which assess essentially basic acoustic changes in music. The performances of the Seashore test progressively decreased from MiAD to MoAD patients, except for pitch interval as others studies have observed, too [10, 43]. The work of Golden et al. [10] also observed an unimpaired sense of pitch interval, but it found a selective deficit of global pitch (melody contour). They suggest that this deficit might be due to auditory working memory deficits and, thus, it might reflect increased demand for coordinated integrative computations between temporo-parietal association cortices vulnerable to AD [44]. The results showed that music aspects of time, rhythm and timbre were more initially resistant to the AD, before dropping to the moderate stage. However, the tonal memory was early altered in the MiAD patients, and then it decreased more in MoAD group. Most of Seashore subtests significantly differentiated the MiAD group from MoAD group, but it did not distinguish the MiAD group from that of the control. The Seashore test would therefore be sensitive to the evolution of the disease, but it could not discriminate effectively the MiAD patients from healthy subjects. In contrast, the extra-linguistic test could distinguish control and MiAD participants, but not between the AD stages. Besides, we found a strong relationship between Seashore and extra-linguistic tests, and so, it lets you choose one of them for evaluations of musical abilities, but taking into account that the extra-linguistic score is composed of more ecological items and faster assessment than those of the Seashore test.

Furthermore, the Seashore and Solfeggio tests showed the strong correlations with the emotional prosody recognition and emotional learning, suggesting that the musical abilities depend on emotional aspects. In our study, emotional prosody can also distinguish control subjects and AD patients, but the most sensitive item to detect MiAD from healthy participants was spontaneous production of intonation. This result suggests the presence of an expressive emotional aprosody from early stage of AD. The similar results were observed by Roberts et al. [45], concerning the early troubles of emotional production, but they also found preserved emotional prosody recognition in patients with AD. Thus, the emotional perceptual aspect would play a more important role in the preservation/impairment of the musical competences than the emotional expressive aspect.

Concerning the musical emotion recognition test, the performances are poorer in AD patients than those of control participants, though the results did not reach to be significantly different between them, despite the cognitive and emotional troubles in the AD groups. Thus, we can observe a trend towards a performance difference between AD and control subjects, though both AD groups showed the similar results. It could also be suggested that processing of musical emotion may be relatively more resistant than other musical skills to AD, as it has previously been observed in other studies [4, 31]. In addition, several studies have also found the similar results using familiar or not familiar music [12, 15, 30, 31]. However, it is necessary to consider a limitation in our work, to the future studies, concerning the small size of items used in this test of musical emotion recognition, because a larger stimulus set with a greater range may have more clearly exposed a deficit. Furthermore, the recognition capacity of musical emotions was not the same for every type of emotion in our study. All participants recognized the joy emotion of music, followed by sadness emotion, but the fear emotion seemed more difficult to recognize from the music pieces by all groups. Fear is a very complex emotional expression and several studies also observed similar results for fear emotion recognition vs. joy and sadness emotions from faces [29, 40, 46]. Recognition impairments for facial expressions of emotion are seen in AD [27, 28] and more altered in semantic dementia. Nevertheless, some studies [4, 46] found unimpaired performances in recognition of non-familiar facial and non-familiar musical emotions in AD.

Moreover, Hsieh et al. [32] observed the common neural substrates supporting the processing of emotions by facial and musical stimuli, involving essentially the right temporal pole, amygdala and insula, but the recognition of musical (but not facial) emotions was also associated with the left anterior and inferior temporal lobe, which are associated with semantics in language. In this sense, the work of Omar et al. [34] observed deficient recognition of emotions from music as well as faces and voices in subjects with frontotemporal lobar degeneration. They observed that the impaired recognition of emotions from music was specifically associated with grey matter loss in a distributed cerebral network including the insula, orbitofrontal cortex, anterior cingulate and medial prefrontal cortex, anterior temporal and more posterior temporal and parietal cortices, amygdala and the subcortical mesolimbic system.

It is also necessary to consider other limitation in our study, concerning the possible semantic associations in some items of musical emotion recognition, for instance, in the case of the fear emotion which can be associated to a movie theme or any other association of an emotion and past experiences. These associations require interactions between musical emotions and music processing. Nevertheless, AD groups showed the important declarative memory troubles. It will be interesting to use a larger item size and to control the semantic associations of musical pieces in future studies analysing musical processing in AD.

Moreover, the preservation/impairment is heterogeneous in the different aspects of musical abilities in AD, essentially due to the diffuse nature of the neural musical network which relies on the participation of the two cerebral hemispheres and in relation to melodic and temporal processing of the functional architecture model by Peretz and Coltheart [47]. The two cerebral hemispheres would be solicited in musical cognition and the preserved or not capacities are indifferent in one or the other. The diffuse nature of the musical neural network would probably be an asset in the relative preservation of certain musical skills. For instance, it is found in the right cerebral hemisphere the time, the timbre, and the joy and sadness recognitions which are mostly preserved at the beginning of the disease. In contrast, the left hemisphere is more responsible for the notions of rhythm, musical reading and the naming and identification of familiar music. This last ability was slightly less affected than other musical skills, maybe due to the relatively good semantic memory and emotional aspect of familiar melodies. We suggested that the emotional perceptive aspect seems to retain certain music skills, which are more resistant than other musical skills to AD. In this sense, several studies have also found an expressive aprosody in the early stages of AD (for review, see [48].

## Conclusions

The present findings have certain practical and clinical implications. This study observed a global deterioration of musical abilities in AD patients. Nevertheless, the performances of musical emotions’ recognition in both AD groups are poorer than those of the control group, but they did not reach statistical significance. Thus, we can suggest that AD currently presents an aphaso-agnoso-apractic-amusia syndrome. Further studies are necessary to improve the limitations observed in this study in order to deep in the musical processing in AD. And future study cohorts should ideally encompass a wider range of AD and other neurodegenerative diseases with longitudinal assessments to determine the sensitivity and specificity of particular musical patterns, associated to histopathological and molecular data. Besides, the present data underline the need to take into account individual variability, which may be amplified by prior musical competence, in order to deeper delineate the alterations in brain mechanisms of music processing produced by AD.

The results of this study also suggest that it is possible to make a fast assessment of the subject’s musical abilities, considering three musical scores: extra-linguistic, Solfeggio and emotional recognition scores. We consider that the Seashore test could be reserved only to deeply complete the musical profile of the subject. Furthermore, our data also suggest that the power of emotional music could enhance the general mental state in a more direct and involuntary neural network, and it could enhance more using music related to the personal experience of the subject. Future studies could find more evidences about the benefits of emotion and music powers on mental health in neurodegenerative diseases, in particular in accessing emotional memories.

## Data Availability

All data and material is available at the Department of Neurology of University Hospital, CHU La Milétrie, Poitiers, France.

## Abbreviations

AD: Alzheimer’s disease
CDR: Clinical Dementia Rating Scale
EL: Educational level
HAD: The Hospital Anxiety and Depression Scale
MiAD: Mild Alzheimer’s disease
ML: Musical level
MMS: Mini-Mental Status Examination
MoAD: Moderate Alzheimer’s disease
